# Serum zonulin level in autistic children and its relation to severity of symptoms a case-control study

**DOI:** 10.1038/s41598-025-11420-0

**Published:** 2025-07-30

**Authors:** Hassan Mohammed Sonbol, Alaa Salah Abdelmawgoud, Nora Marzouk El-kady, Eman Sameh Abdelhay, Hosam Eldin Abdel Tawab

**Affiliations:** 1 Psychiatry Medicine, Faculty of Medicine, Mansoura University, Mansoura, Egypt; 2 Clinical Pathology, Faculty of Medicine, Mansoura University, Mansoura, Egypt; 3 Psychiatric and Mental Health Nursing, Faculty of Nursing, Mansoura University, Mansoura, Egypt

**Keywords:** Autism, Children, Zonulin, Gut-brain axis, Biomarker, Medical research, Paediatric research

## Abstract

Evidence suggests a possible link between Autism spectrum disorder and gut permeability, specifically as indicated by serum zonulin levels. However, limited studies examine this connection to symptom severity, especially in Egypt. Assessing serum zonulin level in children with autism and its relation to the severity of symptoms. In Mansoura University Hospital’s pediatric psychiatry outpatient clinics, case-control research was carried out with children with Autism diagnoses and age- and gender-matched typically developing controls. The Childhood Autism Rating Scale was used to gauge the severity of the symptoms, and the Enzyme-Linked Immunosorbent Assay (ELISA) was used to detect serum zonulin levels. Serum zonulin levels were considerably higher in children with Autism than in the control group (*p* < 0.05). Additionally, there was a positive correlation between higher zonulin levels and the degree of autism symptoms as determined by CARS scores (r = X, *p* < 0.05). Children with severe Autism had the highest zonulin levels, according to subgroup analysis, which suggests a possible connection between gut permeability and the intensity of symptoms. This study emphasizes how serum zonulin may serve as a biomarker for intestinal permeability in kids with Autism and how it may be related to the intensity of symptoms. These results highlight the need for more investigation into the gut-brain axis as a potential therapeutic target for Autism. Addressing gut permeability may provide new ways to lessen the intensity of symptoms and enhance results for children with Autism.

## Introduction

Autism Spectrum Disorder (ASD) is highly variable, as children display a diverse range of behavioral symptoms that differ in type and severity^1^. Twin studies state that ASD has a significant genetic factor, with a heritability rate of up to 87%, although the exact causes remain unidentified^2,3^. Factors such as prenatal and neonatal environmental exposures and complications during delivery, and it has been proposed that advanced paternal age might contribute to the development of ASD^4^.

Increasing interest among physicians has emerged regarding the influence of epigenetics on gene expression in ASD, particularly following extensive research in recent years. This study explores the gut-brain-microbiota axis’s possible role in the disorder’s etiology^5^. Children with ASD are about five times more likely to experience gastrointestinal (GI) tract issues compared to the general population. These GI problems can interfere with sleep and behavioral regulation, potentially worsening mental health comorbidities^6^. The gastrointestinal challenges establish a link between the gut-brain axis and disorders of neurodevelopment despite the uncertainty surrounding the cause-and-effect relationship^7^. Approximately 50% of kids with ASD present with at least one GI symptom^8,9^. Diarrhea and constipation are the most often reported GI disorders in children with ASD, who are more susceptible to these conditions than their neurotypical peers^10,11^. Evidence indicates a correlation between the severity of ASD and the extent of GI symptoms^12,13^.

The data suggest that gastrointestinal problems may influence the development of ASD. Certain bacterial groups, including Proteobacteria, Lactobacillus, Bacteroides, Desulfovibrio, and Clostridium, are found more frequently in autistic individuals, whereas Bifidobacterium, Blautia, Dialister, Prevotella, and Veillonella are consistently less present^14^. Proteobacteria are associated with host inflammation and are prevalent in the gastrointestinal tracts of individuals with autism^15^. Animal studies have shown that lipopolysaccharide (LPS) produced by Proteobacteria may reduce the brain’s levels of the antioxidant glutathione (GSH)^16,17^.

ASD and other gastrointestinal-related gut-brain issues may be influenced by a lack of beneficial bacterial metabolites and excess harmful ones, impairing communication between the gut and brain. The gut-brain axis and the “leaky gut” hypothesis suggest that altered metabolites can enter the bloodstream, impacting neurodevelopment^18^. A recent study has shown that gut microbiota significantly affects the pathophysiology of ASD by influencing the development of the central nervous system, neuropsychology, and gastrointestinal balance through the microbiota-gut-brain axis^19,20^. A recent study indicates a correlation between gastrointestinal tract disorders, dysbiosis, and an elevated risk of central nervous system diseases. Research increasingly suggests that zonulin may significantly contribute to the etiology of some microbial–gut-brain axis illnesses^21^.

The zonulin family’s primary members include structurally and functionally similar peptides such as pre-haptoglobin-2. According to Vanuytsel et al.^22^, Zonulin family peptides, collectively referred to as zonulin, are known physiological modulators of tight intercellular junctions. They regulate the mechanisms governing the intestinal epithelial paracellular pathway. Zonulin appears to be the primary regulator of permeability in both the gut-blood barrier and the blood-brain barrier, and it has been used as a clinical biomarker for intestinal permeability(IP)^23^. Moreover, zonulin supports intestinal innate immunity and may act as an inflammatory biomarker. Increased IP has been linked to adult mental disorders, and this permeability often develops in conjunction with inflammation^24^.

Esnafoglu et al.^25^ have noted that zonulin is associated with autoimmune diseases, chronic inflammation, and autism spectrum disorder (ASD), which may have an autoimmune component. Studies show that children with ASD exhibit heightened immunological sensitivity to certain proteins, such as gluten, in specific grains. This sensitivity may result from increased intestinal permeability^26^. Furthermore, social impairments in ASD have been related to elevated blood zonulin levels compared to control groups^27^. While these studies provide insights into the relationship between zonulin levels and autism, they do not specifically address the Egyptian population. The study on vitamin A supplementation in Egyptian children with ASD highlights the potential for nutritional interventions to impact ASD symptoms. However, it does not address the gut-brain axis and zonulin levels^28^.

While previous studies have examined zonulin in ASD, this study is the first to examine this relationship in the Egyptian population, addressing a significant research gap in Middle Eastern populations. Additionally, the present study provides the first comprehensive correlation analysis between zonulin levels and specific GI symptom subscales using validated instruments, offering novel insights into the gut-brain axis in ASD. Therefore, a focused investigation in Egypt examining serum zonulin levels about autism severity, considering local dietary and environmental factors, could fill this research gap and contribute to a more comprehensive understanding of ASD in different populations.

## Materials & methods

### Setting and duration

This case-control research was conducted at the Psychiatry outpatient clinics for children at Mansoura University Hospital in Egypt from November 2022 to September 2023. These clinics offer complementary therapy for pediatric patients suffering from various mental disorders, examinations, diagnoses, and medication prescriptions. The clinic operates from 8 A.M. to 1 P.M. twice a week. The study was conducted over two working days in a private room at the outpatient clinic to ensure privacy and confidentiality during the interviews.

### Study design

A comprehensive case-control study was undertaken.

### Recruitment process and sample size

The participants in the research were selected using a convenience sampling method. Initially, 98 children diagnosed with autism spectrum disorder following DSM-5 criteria were evaluated^29^. However, due to varying exclusion criteria, 19 infants were deemed unsuitable during the screening process. Additionally, 17 children opted not to participate, and *9 withdrew from the research by not completing all the questionnaires*. Ultimately, the study included 53 participants.

Control participants were recruited from attendees of the Children’s outpatient clinics at Mansoura University Hospitals who were visiting for routine check-ups or minor non-psychiatric conditions. The estimate of the sample size was derived from the blood zonulin levels of children with autism spectrum disorders in comparison to a control group, as mentioned in previous research^30^. The G Power program version 3.1.9.7 was used to determine the sample size. The emphasis was placed on an effect size of 0.66, and a two-tailed test with an alpha error of 0.05 and a power of 90% was utilized. According to the findings of this research, the minimum number of participants necessary for each group would be 53.

### Exclusion criteria of the case group

Children with a history of probiotic usage, gluten-free or casein-free diets, or any other specialized diet within the last year were excluded from the study. Additionally, children who were obese (BMI > 95th percentile for age and sex) or diagnosed with gastrointestinal, allergic, inflammatory, or autoimmune conditions were excluded. Participants testing positive for celiac antibodies were also removed from the study.

### Exclusion criteria of the control group

Control participants were recruited specifically for this study from the same hospital setting. The exclusion criteria included children with documented psychiatric or neurological disorders, physical, mental, or linguistic developmental delays, learning disabilities, and those under 3 years of age.

### Tools

Study participants received psychiatric interviews conducted by two distinct psychiatrists before being sent to clinical psychologists. A thorough evaluation methodology for autism was implemented, including:

Tool I: Sociodemographic and Clinical Sheet: This tool sought to collect data on the children’s age, gender, residence, and family economic position^31^. Urban residence was defined as living in Mansoura city proper, while rural residence included surrounding villages and agricultural areas. Socioeconomic status was assessed using a validated Egyptian socioeconomic scale that considered education, occupation, and family income. Furthermore, the psychological evaluation of IQ used the DSM-5 criteria for intellectual impairment^32^.

Tool II: diagnosis and severity evaluation of autism utilizing the Arabic version of the Childhood Autism Rating Scale (CARS), which encompasses fifteen domains of autistic behavior in children (scores: 1–4 for each item, ranging from standard to severely abnormal). On a scale of 1 to 60, total scores range from less than 30 to 60. Scores below 30 indicate a non-autistic categorization, scores between 30 and 36.5 indicate mild to moderate autism, and scores between 37 and 60 indicate severe autism^33^. In this study, we use the Arabic version^34^.

Tool III: the Gilliam Autism Rating Scale (GARS), consisting of fifty-six items to assess the severity of autism, where a higher score indicates a more severe autistic condition^35^. We used the GARS Arabic version^36^.

Tool IV: The Gastrointestinal Symptom Rating Scale GSR scale with parental assistance^37^. The control group was not given this questionnaire. The questionnaire had 15 distinct symptoms, each accompanied by an individual subscale. Each question was assessed on a scale from 0 to 3 points. Typical symptoms were getting 0 points, whereas symptoms that differed from the average were assigned 3 points. The questionnaire had a minimum possible score of 0 and a maximum possible score of 45 points. In order to reevaluate the questions, they were divided into three categories: lower gastrointestinal symptoms (flatus, altered stool frequency, stool consistency, urgent defecation need, and inadequate sensation of discharge), upper gastrointestinal symptoms (belching, heartburn, reflux, and nausea/vomiting), and general symptoms (restlessness, abdominal pain, distension, and rumbling)^38^.

Tool V: MINI-KID was developed in collaboration with psychiatrists and physicians in the United States and Europe. This brief semi-structured interview is intended to diagnose children between the ages of 4 and 17^31^. The test evaluates 24 mental illnesses as defined by the DSM-IV and ICD-10. The tool’s reliability is further supported by studies showing its effectiveness in diagnosing co-occurring psychiatric disorders in conditions like autism spectrum disorder (ASD) and tuberous sclerosis complex (TSC), where high rates of comorbidity were observed^39,40^.

### Procedure

After receiving the study permission from the head department of psychiatry at Mansoura University and the acceptance by the Faculty of Medicine Ethical Committee, informed written permission was acquired from the parents before the children were included in the study. All children were diagnosed according to the DSM-5 criteria for autism. Children with ASD were evaluated using a GSR scale with parental assistance. The control group was not administered this questionnaire since it included patients without a GIS complaint. Patients in the high and low-intensity GIS symptom groupings were referred for additional testing based on their mean scores, which were calculated. Autism Spectrum Disorder (ASD) cases were assessed using the Childhood Autism Rating Scale (CARS) and MINI-KID.

A five ml blood sample was collected from each patient and control group to assess serum zonulin levels. Centrifugation at 4000 rpm for 10 min was employed to isolate the serum, which was subsequently stored at -80 °C until further analysis. A sandwich enzyme-linked immunosorbent assay (ELISA) utilizing a Human Zonulin ELISA kit (EASTBIOPHARM, Hangzhou Eastbiopharm Co. Ltd., China, Lot No: 20170421) was implemented to quantify serum zonulin concentrations.

### Ethical consideration

The research ethical board of Mansoura University’s Faculty of Medicine (23.05.2173) approved the study protocol, and the managers of the medical facilities where the study was carried out also approved it. Each participant provided written, informed consent, guaranteeing that privacy and confidentiality would be upheld during the research. The information was gathered only to be utilized for this study. Participants were also told they could leave the research at any moment without applying any penalty.

### Statistical analysis

Data were analyzed with SPSS version 22. Qualitative data were expressed as numerical values and percentages, and quantitative data were subjected to normality assessment using the Shapiro-Wilk test. The mean and standard deviation represented data that followed a normal distribution, whereas data that did not conform to a normal distribution were displayed as median and range. Statistical tests were performed according to the data type. The Chi-Square test was used for categorical data analysis. Spearman or Pearson correlation was used to evaluate the associations between continuous variables.

## Results

**Fig. 1:**
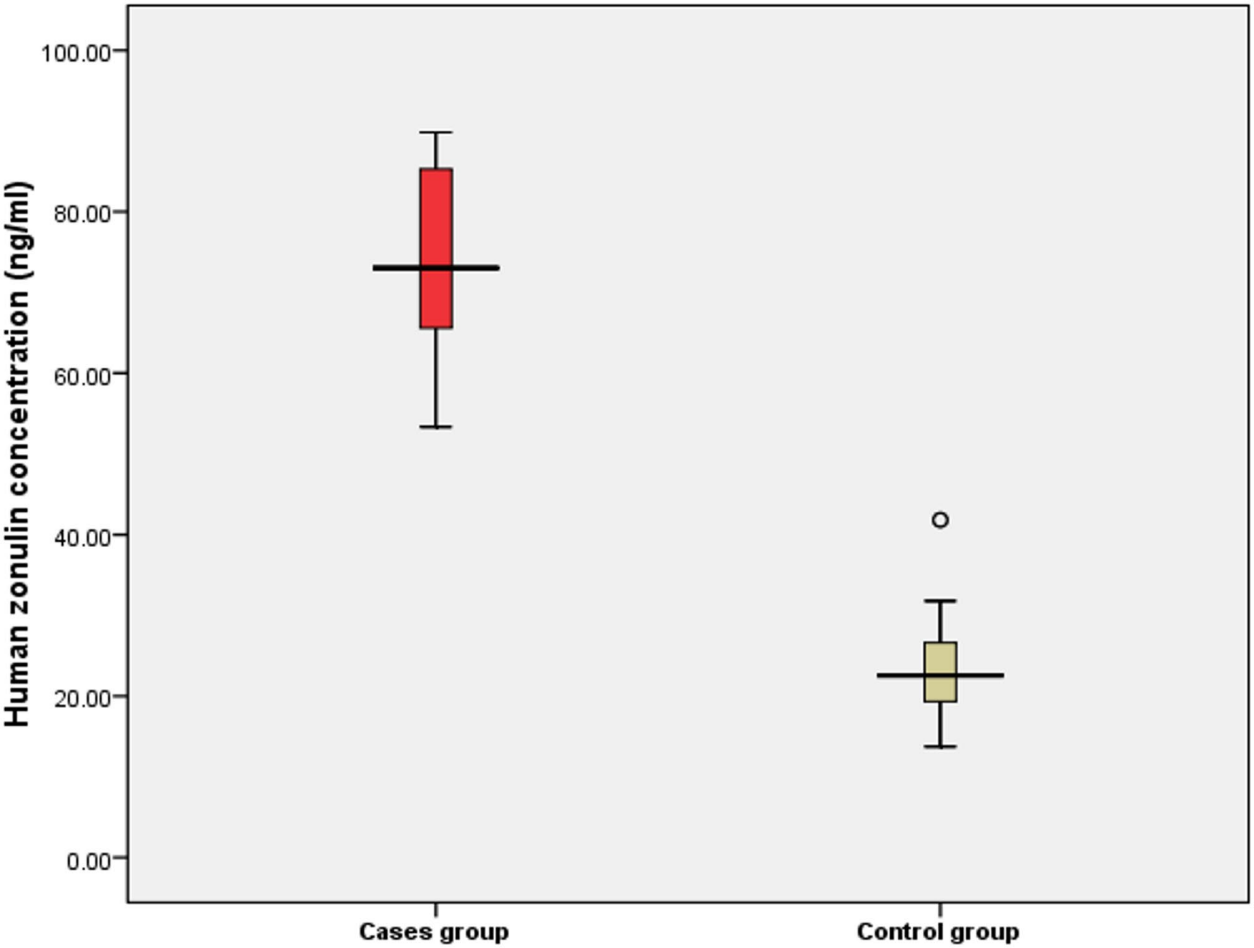
Box& Whisker plot showing median human Zonulin among studied groups.

The study included 53 children with ASD (mean age: 7.87 ± 1.75 years) and 53 age- and gender-matched typically developing controls (mean age: 8.21 ± 1.70 years) Table 1. No significant difference is detected between cases with autism and control groups as regards age, sex, residence, and socioeconomic status. A statistically significant lower mean IQ among cases than the control group (56.77 &90.88, respectively). Median human zonulin is higher among cases than in control groups (73.04 & 22.54 ng/ml, respectively), as shown in Table 1; Fig. 1.

**Table 1:** Comparison of socioeconomic characteristics between studied groups.

Socioeconomic characteristics	Cases N = 53	Control group N = 53	Test of significance
Age/years	7.87 ± 1.75	8.21 ± 1.70	T = 1.01
Mean ± sd			*P* = 0.314
Sex			
Male	39(73.6)	37(69.8)	χ^2^ = 0.186
Female	14(26.4)	16(30.2)	*P* = 0.666
Residence			
Urban	24(45.3%)	20(45.5%)	χ^2^ = 0.0
Rural	29(54.7%)	24(54.5%)	*P* = 0.987
Socioeconomic status			
Low	20(37.7%)	17(38.6%)	χ^2^ = 0.160
Average	27(50.9%)	21(47.7%)	*P* = 0.923
High	6(11.3%)	6(13.6%)	
Iq	56.77 ± 12.52	90.88 ± 5.11	T = 18.37
Mean ± sd			*P* < 0.001*
Human zonulin (ng/ml)	74.01 ± 11.01	23.59 ± 6.14	T = 29.11
Mean ± sd Median (min–max)	73.04(53.31–89.84)	22.54(13.72–41.83)	*P* < 0.001*

T: student t test, χ^2^=chi-square test. * Statistically significant at *p*  ≤  0.05.

*Urban: living in Mansoura city, Rural: surrounding villages and agricultural areas.

**Fig. 2:**
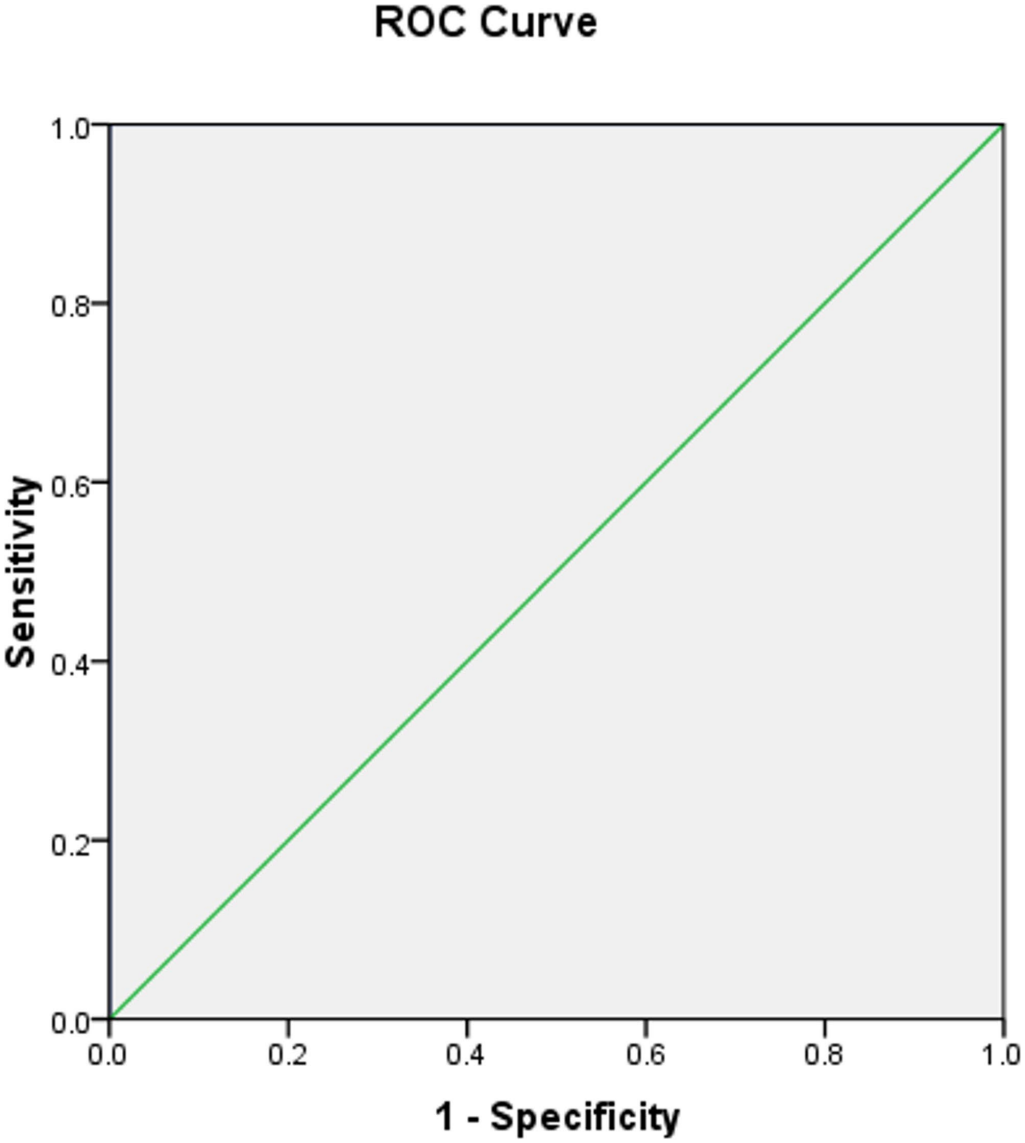
ROC curve of Human Zonulin in differentiating between cases & and control groups.

Receiver operating characteristics have been done for human zonulin in differentiating between cases and control groups and were excellent, with the best-detected cut-off point > 47.57 yielding sensitivity of 100%, specificity of 100%, and total accuracy of 100% as shown in Table 2; Fig. 2.

**Table 2:** Validity of human Zoulin in differentiating between cases & control groups.

	AUC (95%CI)	*P* value	Cut off point	Sensitivity %	Specificity %	PPV%	NPV%	Accuracy %
Human zonulin (ng/ml)	1.0 (1.0–1.0)	< 0.001*	> 47.57	100.0	100.0	100.0	100.0	100.0

* Statistically significant at *p* ≤  0.05.

AUC: Area under curve, PPV: Positive predictive value, NPV: Negative predictive value.

**Fig. 3:**
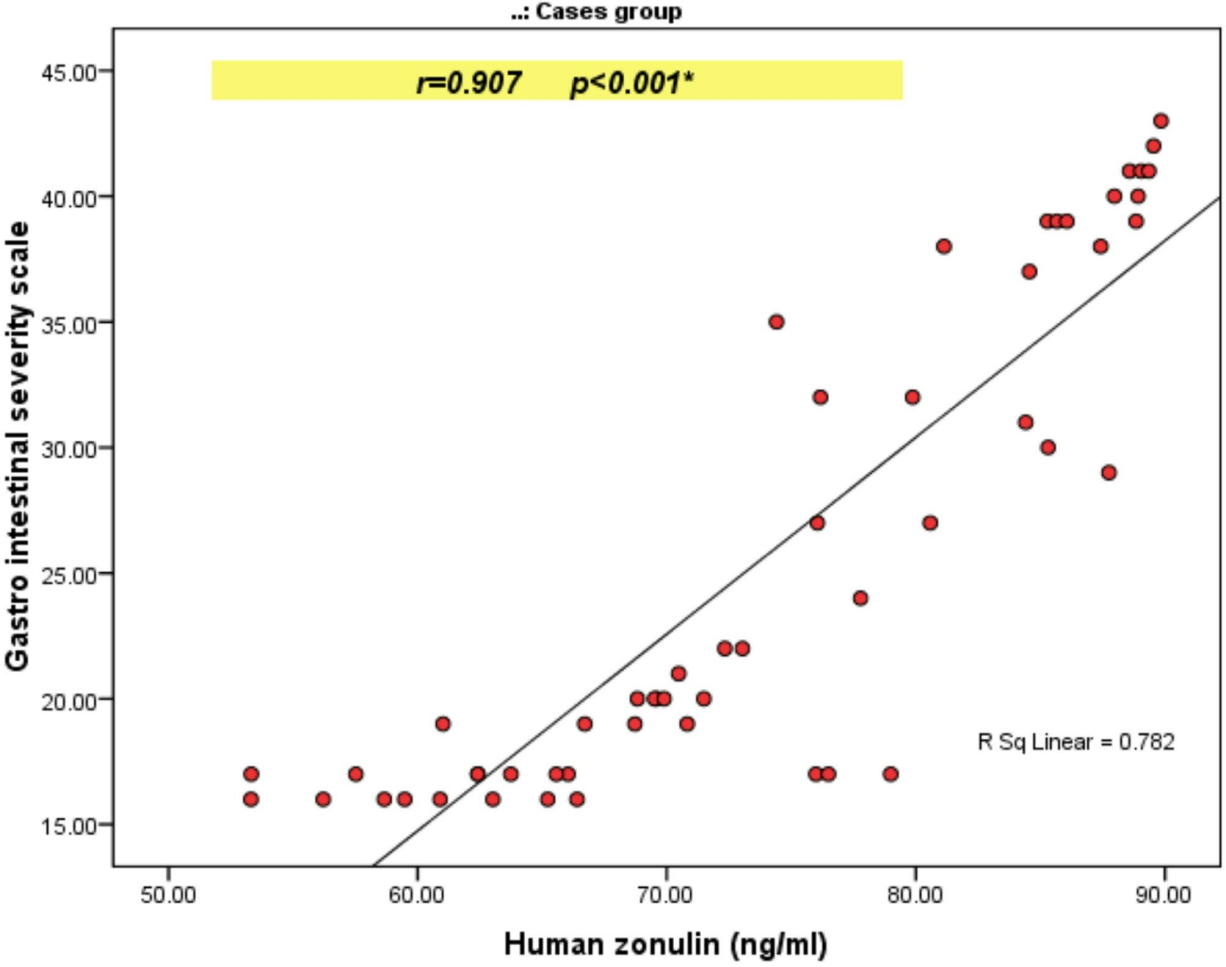
Scatter diagram showing a correlation between human zonulin and gastrointestinal severity score among studied cases with an autism spectrum disorder.

A statistically significant negative correlation between human zonulin and IQ (*r*=-0.754, *P* < 0.001). A statistically significant positive correlation between human zonulin and the following: CARS(*r* = 0.812), CARS severity of autism(*r* = 0.846), and Gilliam scores (*r* = 0.635), as shown in Tables 3 and 4. A statistically significant positive correlation between human zonulin and the following; Gastrointestinal severity scale(*r* = 0.907), pain(*r* = 0.768), distention(*r* = 0.644), sucking sensation(*r* = 0.487), rumbling abdomen(*r* = 0.571), score of the general subscale(*r* = 0.834), nausea, vomiting (*r* = 0.641), heartburn(*r* = 0.717), acid regurgitation( *r* = 0.729), score of the upper GIT subscale(*r* = 0.780), increased flatus(*r* = 0.098), decreased passage of stool(*r* = 0.313), increased passage of stool(*r* = 0.273), loose stool(*r* = 0.318), hard stool(*r* = 0.412), urgency of defecation(*r* = 0.297), sensation of incomplete defecation( *r* = 0.366), and score of lower GIT subscale(*r* = 0.648) as shown in Table 5; Figs. 2 and 3.

**Table 3:** Correlation between human Zonulin (ng/ml) and IQ. Age and socioeconomic status.

	Human zonulin (ng/ml)	
	R	P value
IQ	-0.754	< 0.0001*
Age( years)	-0.069	0.624
Sex	-0.045	0.985
Socio-economic level	-0.167	0.278

r: Spearman correlation coefficient, * Statistically significant at *p*  ≤  0.05.

**Table 4:** Correlation between human Zonulin (ng/ml) and probability of autism scores among studied cases.

	Human zonulin (ng/ml)	
	R	P value
CARS	0.812	< 0.001*
CARS severity of autism	0.846	< 0.001*
GILLIAM	0.763	< 0.001*
Gilliam probability of autism	0.253	0.068
Gilliam need for support	0.635	< 0.001*

r: Spearman correlation coefficient, *Statistically significant at *p* ≤  0.05.

**Table 5:** Correlation between human Zonulin (ng/ml) and gastro intestinal severity scale and its subdomains among studied cases.

Items	Human zonulin (ng/ml)	
	*R*	*P value*
Gastro intestinal severity scale	0.907	< 0.001*
Pain	0.768	< 0.001*
Distention	0.644	< 0.001*
Sucking sensation	0.487	< 0.001*
Rumbling abdomen	0.571	< 0.001*
Score of the general subscale	0.834	< 0.001*
Nausea, vomiting	0.641	< 0.001*
Heart burn	0.717	< 0.001*
Acid regurgitation	0.729	< 0.001*
Eructation	0.208	0.136
Score of upper GIT subscale	0.780	< 0.001*
Increased flatus	0.098	0.484
Decreased passage of stool	0.313	0.024*
Increased passage of stool	0.273	0.048*
Loose stool	0.318	0.02*
Hard stool	0.412	0.002*
Urgency of defecation	0.297	0.03*
Sensation of incomplete defecation	0.366	0.007*
Score of lower GIT subscale	0.648	< 0.001*

r: Spearman correlation coefficient, *Statistically significant at *p* ≤  0.05.

**Fig. 4:**
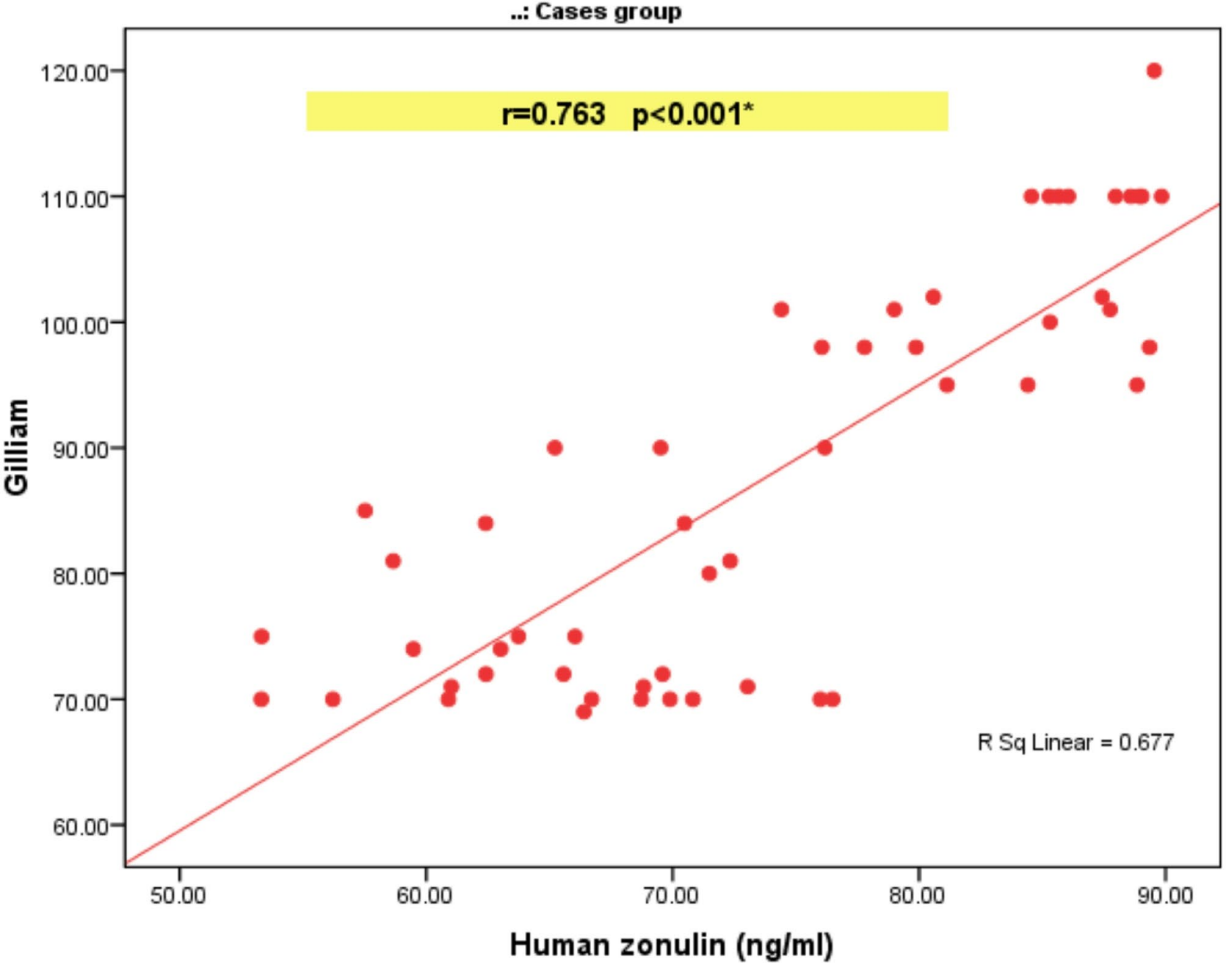
Scatter diagram showing a correlation between human zonulin & gilliam among studied ASD cases.

**Fig. 5:**
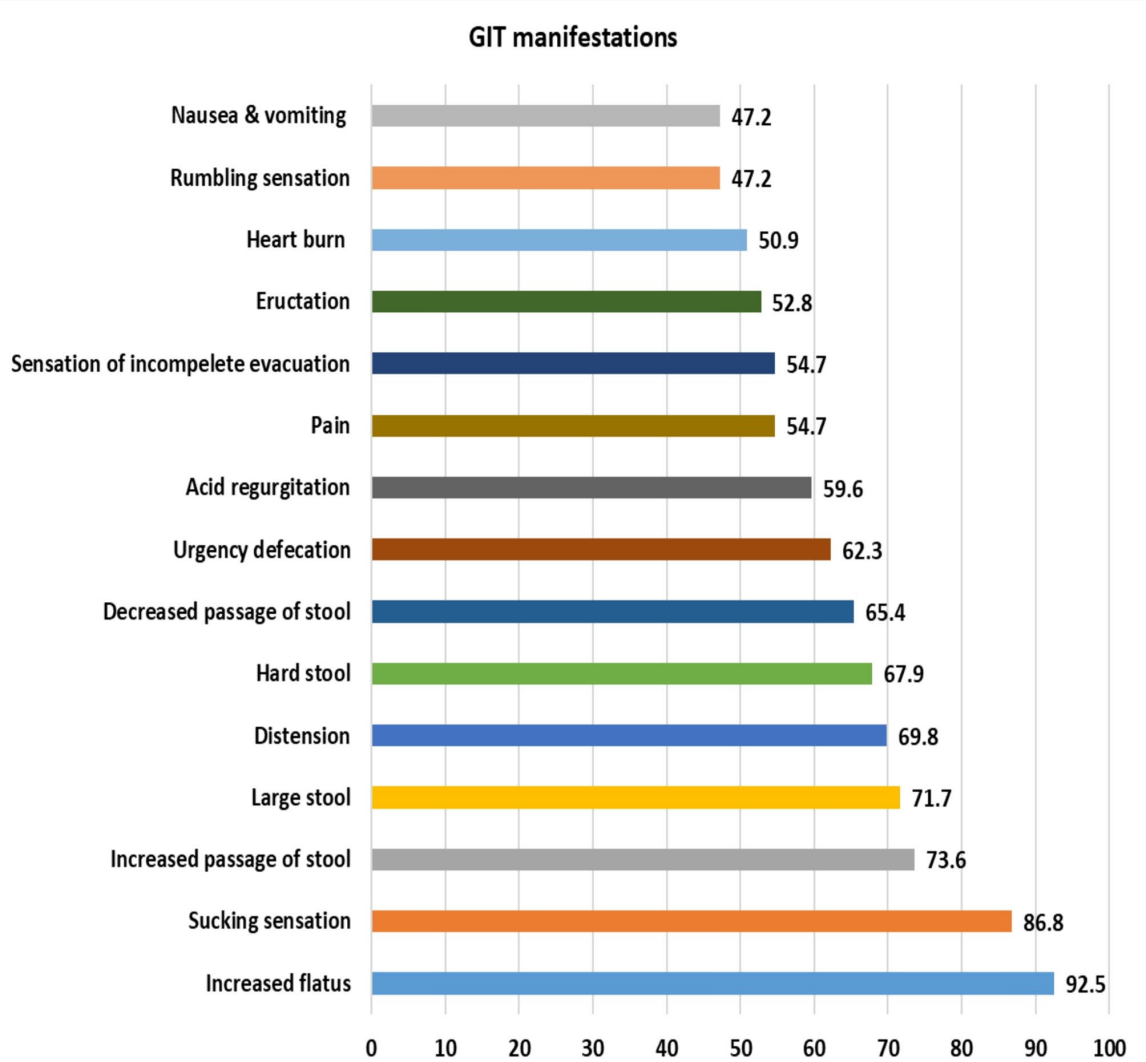
Distribution of the studied cases according to GIT manifestations.

Figure 4 demonstrates a strong, positive linear association between serum human zonulin concentrations and Gilliam scores in the autism cohort. As zonulin levels rise from roughly 50 to 90 ng/ml, Gilliam’s ratings climb in parallel from about 60 to 120, with the fitted regression line capturing this upward trend. The Pearson correlation coefficient is *r* = 0.763, indicating a strong relationship and highly significant association (*p* < 0.001). The coefficient of determination (R² ≈ 0.68) shows that nearly two-thirds of the variability in Gilliam scores can be explained by differences in zonulin, suggesting that gut-derived permeability marker zonulin may be closely linked to the severity of behavioral symptoms measured by the Gilliam scale in these autistic cases (Fig. 5). Figure 5 illustrates the most prevalent GIT manifestations were increased flatus (92.5%) and sucking sensation (86.8%), followed by increased passage of stool (73.6%) and large stool (71.7%). Mid‑range complaints included distension (69.8%), hard stool (67.9%), and decreased passage of stool (65.4%).

A statistically significant higher mean human zonulin among cases with severe autism by CARS scale. A statistically significant higher mean human zonulin among cases needs much more support by Gilliam need for support. A statistically significant higher mean of human zonulin was found among cases with a high degree of GIT symptoms than cases with a low degree of GIT symptoms, as shown in Table 6.

**Table 6:** Association between human Zonulin (ng/ml) and CARS severity, probability of autism and Gastrointestinal severity scale.

	Total number	Human zonulin (ng/ml) mean ± SD	*P* value
CARS severity of autism			
Mild to moderate	30	65.84 ± 6.45	t = 11.78
Severe	23	84.67 ± 4.69	*P* < 0.001*
Gilliam probability of autism			
Possible autism	9	67.69 ± 7.22	t = 1.94
Very likely autism	44	75.30 ± 11.27	*p* = 0.058
Gilliam need for support			
Need little support	10	66.54 ± 7.72	F = 21.84
Need more support	28	70.44 ± 9.73	*P* < 0.001*
Need much more support	15	85.64 ± 4.42	
Gastro intestinal severity scale			
Low degree of GIT symptoms	28	65.46 ± 6.60	t = 10.63
High degree of GIT symptoms	25	83.59 ± 5.72	*p* < 0.001*

t: Student t test, F:One Way ANOVA test, * Statistically significant at *p* ≤ 0.05.

## Discussion

This study demonstrates significantly elevated serum zonulin levels in children with ASD compared to controls, with strong correlations between zonulin levels and autism severity measures. The findings provide important evidence for the gut-brain axis hypothesis in ASD, particularly in the understudied Egyptian population. The most striking finding was the perfect diagnostic accuracy achieved (100% sensitivity and specificity), with children with ASD showing substantially higher zonulin levels (median 73.04 ng/ml) compared to controls (22.54 ng/ml).

### Zonulin as a biomarker of intestinal permeability in ASD

The study results align with and extend previous research on intestinal permeability in ASD. The strong positive correlations between zonulin levels and CARS scores (*r* = 0.812) and Gilliam scores (*r* = 0.763) suggest intestinal permeability directly relates to autism severity, providing objective biochemical evidence for this relationship. These findings are consistent with those of Esnafoglu et al.^25^, who reported elevated zonulin levels in children with ASD and positive correlations with CARS scores. However, our study achieved superior diagnostic discrimination and found no age-related effects on zonulin levels, possibly reflecting population-specific differences or methodological variations. Unlike Jozefczuk et al.^26^, who identified age-related zonulin variations suggesting intestinal barrier immaturity in younger children, we did not observe such patterns in the present cohort. The animal research findings conducted by Watts et al.^41^ provide a different perspective from present human study results. Their study in diabetic rats showed age-related changes in zonulin levels, which differs from the current findings in children with ASD, highlighting the need for human-specific research in this area.

The perfect diagnostic accuracy observed in the present study represents a significant advancement over previous intestinal permeability assessments. Earlier studies using lactulose/mannitol testing showed mixed results, with d’Eufemia et al.^42^ finding increased intestinal permeability in only half of autistic children, while Robertson et al.^43^ found no significant differences between ASD and control groups. The present zonulin-based approach appears to provide more consistent and reliable results, potentially offering a more sensitive biomarker for gut barrier dysfunction in ASD.

### Gastrointestinal symptoms and gut-brain axis dysfunction

The high prevalence of gastrointestinal symptoms in the present ASD cohort (92.9% general GI symptoms, 98% lower GI symptoms) is consistent with established literature reporting GI issues in 9–91% of children with ASD, depending on the study methodology^11^. Study findings align with Ahmed et al.^44^, who reported GI symptoms in 50% of ASD children, with constipation being the most common symptom. The prevalence of GI symptoms in ASD children is approximately 4 times higher than in typically developing children^45^.

Critically, the study finds a strong correlation between zonulin levels and GI symptom severity (*r* = 0.907), which provides mechanistic insight into this relationship. This suggests that increased intestinal permeability directly contributes to gastrointestinal dysfunction in ASD, potentially creating a pathological cycle where gut inflammation increases permeability, allowing passage of inflammatory mediators that could affect brain function and behavior. This mechanistic understanding builds upon the pioneering work of Goodwin et al.^46^, who first highlighted the brain-gut connection in autistic patients exhibiting cerebral dysfunction and malabsorption. The present quantitative zonulin measurements provide molecular evidence for this connection, demonstrating that gut barrier dysfunction’s severity correlates with gastrointestinal symptoms and core autism behaviors.

These findings support the growing evidence for gut microbial dysbiosis in ASD, defined as an imbalance in gut microbiota organisms, which has been reported in numerous investigations of children with ASD^45^. The relationship between increased intestinal permeability and autism severity, as demonstrated by the present study’s strong correlations, suggests a direct mechanistic link between gut dysfunction and behavioral symptoms.

The present findings have significant therapeutic implications, particularly regarding dietary interventions commonly used in ASD management. The elevated zonulin levels observed in the current study (median 73.04 ng/ml) provide a measurable biomarker that could potentially monitor the effectiveness of dietary interventions.

The GFCF diet, based on the “opioid-excess theory,” aims to reduce intestinal permeability by eliminating gluten and casein^47,48^. This theory assumes that some ASD children have insufficient enzyme production for digesting gluten and casein-related foods, combined with increased gut permeability. Large peptides derived from gluten and casein are incompletely converted to amino acids and, with increased gut permeability, can enter the bloodstream and reach the brain by crossing the blood-brain barrier. Their binding to opioid receptors potentially produces autism symptoms^49^.

The current zonulin findings provide biological plausibility for this approach. Studies in celiac disease have shown that gluten-free diets reduce zonulin levels^50,51^, and De Magistris et al.^52^ observed that intestinal permeability was elevated in 30% of autistic children and 20% of their first-degree relatives, with improvements seen following gluten- and casein-free diets. Given the demonstration of markedly elevated zonulin levels in ASD children, similar dietary interventions might be expected to normalize these levels.

The clinical evidence supporting GFCF diets is compelling. Dosti, et al.^53^, and Cade et al.^54^ reported 87% of children with autism had IgG antibodies to gluten compared to 1% of controls, and 90% had antibodies to casein compared to 7% of controls. In their follow-up study involving 70 autistic children maintained on GFCF diets for 1–8 years, 81% showed significant improvement in social isolation, eye contact, mutism, learning skills, hyperactivity, stereotypic activity, and panic attacks by the third month, with improvements continuing over the next 12 month s^54^. Present zonulin measurements could provide an objective biomarker to monitor such interventions, addressing the reliance on subjective behavioral assessments.

Beyond dietary approaches, the study findings suggest that direct zonulin inhibition could represent a novel therapeutic strategy. Clinical investigations in celiac disease patients have shown that the zonulin inhibitor larazotide acetate can alleviate gastrointestinal and non-gastrointestinal symptoms^55,56^. Heberling et al.^57^suggested that investigating zonulin inhibitors for the treatment of autism spectrum disorders may be beneficial. Given our demonstration of elevated zonulin levels correlating with autism severity, such zonulin inhibitors warrant investigation in ASD populations.

Long-term studies provide additional context for the present findings. Ibrahim et al.^58^ conducted a 21-year longitudinal study monitoring GI symptoms in 124 children with ASD, finding that while overall GI symptom frequency was not significantly different from controls, the rates of nutritional problems and constipation were notably higher in the ASD cohort. During follow-up, diarrhea (50.3%) and constipation (33.9%) rates increased, with restlessness (80.3%) and increased flatus (85.7%) being the most common symptoms. These findings underscore the chronic nature of GI dysfunction in ASD and support the need for objective biomarkers like zonulin to monitor treatment response over time.

Adams et al.^59^ demonstrated a substantial positive correlation between Autism Treatment Evaluation Checklist (ATEC) scores and gastrointestinal symptoms (*r* = 0.60, *p* < 0.001), supporting the current finding of strong correlations between autism severity and GI dysfunction. The study’s stronger correlation coefficient (*r* = 0.907 between zonulin and GI symptoms) suggests that zonulin may be a more sensitive marker of the gut-brain connection than subjective symptom assessments alone.

### Limitations and future directions

While the study provides robust evidence for elevated zonulin levels in ASD, several limitations should be acknowledged. Longitudinal studies are needed to establish whether elevated zonulin levels precede autism symptom development or result from it.

Future studies should consider stratifying autism subgroups based on GI symptom profiles to better understand the zonulin-autism relationship across different phenotypes. The present study provides preliminary evidence for this association. However, larger studies examining specific autism subgroups with distinct GI presentations would enhance the understanding of the gut-brain axis in ASD. Further research should investigate whether dietary or pharmacological interventions that normalize zonulin levels lead to corresponding improvements in autism symptoms. Additionally, the relationship between zonulin levels and specific autism symptom domains (social communication, restricted interests, sensory issues) warrants detailed investigation.

## Conclusions

The present study provides compelling evidence that serum zonulin levels are significantly elevated in children with ASD and correlate strongly with autism severity and gastrointestinal symptoms. These findings support the gut-brain axis hypothesis in ASD pathophysiology and suggest that zonulin could serve as both a diagnostic biomarker and a therapeutic target. The perfect diagnostic accuracy achieved and strong correlations with clinical measures position zonulin as a valuable objective measure in ASD research and potentially in clinical practice.

The therapeutic implications are substantial. They provide a biological rationale for dietary interventions while suggesting novel pharmacological approaches targeting intestinal permeability. As we move toward personalized medicine approaches in ASD, zonulin measurements could help identify children most likely to benefit from gut-targeted interventions, ultimately improving outcomes for this vulnerable population.

### Implications

This study’s results have significant implications for clinical practice and future research. The notable correlation between serum zonulin levels and symptom severity in autistic children indicates that zonulin may function as a biomarker for evaluating the severity of autism spectrum disorder (ASD). Psychiatrists may investigate blood zonulin levels to assess illness progression or therapy response, enhancing personalized care options. These findings underscore the need for more research on gut-brain interactions in ASD, given that raised zonulin levels correlate with heightened intestinal permeability, potentially affecting neurological development. Future research should prioritize more considerable, longitudinal investigations to elucidate the causal association between zonulin and autistic symptoms and investigate possible treatment strategies targeting the modulation of zonulin levels or gut permeability. Furthermore, integrating broader demographic variables and examining other possible biomarkers might improve the comprehension of autism’s intricate etiology.

## Data Availability

Data will be available upon reasonable request from the corresponding author.

## Abbreviations

ASD: Autism Spectrum Disorder
CARS: Childhood Autism Rating Scale
GARS: Gilliam Autism Rating Scale
ELISA: Enzyme-Linked Immunosorbent Assay
MINI-KID: Mini International Neuropsychiatric Interview for children and adolescents
GI: Gastro Intestinal
IP: Intestinal Permeability
